# The importance of the “how”: the case for differentiated service delivery of long‐acting and extended delivery regimens for HIV prevention and treatment

**DOI:** 10.1002/jia2.26095

**Published:** 2023-07-13

**Authors:** Anna Grimsrud, Lynne Wilkinson, Sinead Delany‐Moretlwe, Peter Ehrenkranz, Kimberly Green, Maureen Murenga, Kenneth Ngure, Nelson J. Otwoma, Nittaya Phanuphak, Wim Vandevelde, Marco Vitoria, Helen Bygrave

**Affiliations:** ^1^ HIV Programmes and Advocacy IAS – the International AIDS Society Cape Town South Africa; ^2^ Centre for Infectious Disease Epidemiology and Research University of Cape Town Cape Town South Africa; ^3^ Wits RHI University of the Witwatersrand Johannesburg South Africa; ^4^ Global Health Bill & Melinda Gates Foundation Seattle Washington USA; ^5^ Primary Health Care PATH Seattle Washington USA; ^6^ Primary Health Care PATH Hanoi Vietnam; ^7^ Lean on Me Foundation Nairobi Kenya; ^8^ School of Public Health Jomo Kenyatta University of Agriculture and Technology Nairobi Kenya; ^9^ Department of Global Health University of Washington Seattle Washington USA; ^10^ National Empowerment Network of People Living with HIV/AIDS in Kenya (NEPHAK) Nairobi Kenya; ^11^ The Thai Red Cross AIDS Research Centre – PREVENTION Bangkok Thailand; ^12^ Global Network of People Living with HIV (GNP+) Cape Town South Africa; ^13^ Global HIV, Hepatitis, and Sexually Transmitted Infections Programmes World Health Organization Geneva Switzerland; ^14^ IAS ‐ International AIDS Society Geneva Switzerland

**Keywords:** differentiated service delivery, long‐acting extended delivery, HIV prevention, HIV treatment, ARV, health systems

## Abstract

**Introduction:**

Long‐acting and extended delivery (LAED) regimens for HIV treatment and prevention offer unique benefits to expand uptake, effective use and adherence. To date, research has focused on basic and clinical science around the safety and efficacy of these products. This commentary outlines opportunities in HIV prevention and treatment programmes, both for the health system and clients, that could be addressed through the inclusion of LAED regimens and the vital role of differentiated service delivery (DSD) in ensuring efficient and equitable access.

**Discussion:**

The realities and challenges within HIV treatment and prevention programmes are different. Globally, more than 28 million people are accessing HIV treatment—the vast majority on a daily fixed‐dose combination oral pill that is largely available, affordable and well‐tolerated. Many people collect extended refills outside of health facilities with clinical consultations once or twice a year. Conversely, uptake of daily oral pre‐exposure prophylaxis (PrEP) has consistently missed global targets due to limited access with high individual cost and lack of choice contributing to substantial unmet PrEP need. Recent trends in demedicalization, simplification, additional method options and DSD for PrEP have led to accelerated uptake as its availability has become more aligned with user preferences. How people currently receive HIV treatment and prevention services and their barriers to adherence must be considered for the introduction of LAED regimens to achieve the expected improvements in access and outcomes. Important considerations include the building blocks of DSD: who (provider), where (location), when (frequency) and what (package of services). Ideally, all LAED regimens will leverage DSD models that emphasize access at the community level and self‐management. For treatment, LAED regimens may address challenges with adherence but their delivery should provide clear advantages over existing oral products to be scaled. For prevention, LAED regimens expand a potential PrEP user's choice of methods, but like other methods, need to be delivered in a manner that can facilitate frequent re‐initiation.

**Conclusions:**

To ensure that innovative LAED HIV treatment and prevention products reach those who most stand to benefit, service delivery and client considerations during development, trial and early implementation are critical.

## 1 INTRODUCTION

Successes in the HIV response can be attributed to parallel innovations both in the development of antiretroviral drugs for treatment and prevention, the “what,” as well as in the delivery of these commodities, the “who,” “where” and “when.” In HIV treatment, the development of oral dolutegravir‐based regimens [1] has been so well‐received that more than 80% of people globally now take the same one‐pill‐a‐day regimen (tenofovir‐lamivudine‐dolutegravir or TLD) regardless of where they live. Similarly, ground‐breaking was the discovery of effective biomedical solutions for preventing HIV acquisition in the form of pre‐exposure prophylaxis (PrEP), which has seen delays in reaching similar uptake [2, 3, 4, 5].

Beyond progress in the development of antiretroviral drugs and their formulations, the HIV response has pioneered innovations in service delivery. Since 2015, the World Health Organization (WHO) has recommended a differentiated service delivery (DSD) approach—acknowledging the diverse needs of people living with and vulnerable to HIV [6, 7]. A DSD approach considers the “building blocks” or the “who,” “where,” “when” and “what” of service delivery and adapts these to meet the needs and expectations of clients while acknowledging the resource constraints of the public health system [8, 9].

Daily oral pills have been the primary formulation of antiretroviral therapy (ART) for both HIV prevention and treatment. There is growing recognition of the potential role of long‐acting and extended delivery (LAED) regimens [10, 11, 12] with recent approvals for a long‐acting injectable antiretroviral combination for treatment [13] and a long‐acting vaginal ring and a long‐acting injectable [14] for prevention. LAEDs offer unique benefits to expand uptake, effective use and adherence. LAEDs should follow the example of TLD by tying product development to the creation of target product profiles that address critical service delivery and adherence challenges. Beyond clinical science outcomes, implementation research to address programmatic considerations will be essential [15]. A safe and efficacious LAED will only be impactful if it can be delivered with DSD approaches that balance system constraints against client needs, often defined by the outcomes of acceptability, feasibility, effectiveness, cost and appropriateness [16, 17]. In particular, consideration of whether a LAED may address some of the behavioural challenges to adherence (e.g. combatting stigma and challenges to disclose) should be considered.

Future LAED products for HIV treatment and prevention include a number of potential delivery mechanisms or modalities: longer‐acting oral tablets; intra‐muscular, sub‐cutaneous and self‐injections; implants and different types of patches. In this commentary, we outline the role of DSD in the provision of these future and diverse LAED products for HIV treatment and prevention.

## 2 DISCUSSION

The realities and challenges within HIV treatment and prevention programmes are distinctive, with marked differences in access and scale‐up of ART compared to the limited scale‐up seen for PrEP. As a result, the future positioning of LAED products and their integration into DSD models is likely to differ as they address these distinct challenges. Table 1 outlines suggested building blocks of DSD for both treatment and prevention considering DSD models with daily oral tablets, DSD with current LAED and ideal service delivery with future available long‐acting formulations.

**Table 1:** Ideal differentiated service delivery models with future long‐acting extended delivery antiretrovirals for HIV treatment and prevention

	HIV antiretroviral treatment			HIV antiretroviral prevention		
Building blocks of service delivery	Current DSD model with daily oral tablets	DSD with current LAED^a^	Ideal with future LAEDs (including oral tablets, injections, implants and patches)	Current DSD model with daily oral tablets	DSD with current LAED^b^	Ideal with future LAEDs (including oral tablets, injections, implants and patches)
WHEN Service frequency (frequency of refills and clinical consultations)	3‐ to 6‐monthly drug pickup 6‐ to 12‐monthly clinical visits	1‐ to 2‐monthly IM injections with 6‐ to 12‐monthly clinical visits,^c^ 6‐monthly SC injection with 6‐ to 12‐monthly clinical review^d^	6‐ to 12‐monthly delivery system (LA oral tablets, longer IM injections, SC self‐injections, implants and patches) 6‐ to 12‐monthly clinical visits Alignment with visits for other medical needs	3‐monthly PrEP refills (moving towards 6 monthly) 3‐ to 6‐monthly clinical visits	2‐monthly IM injections and clinical visits	6‐ to 12‐monthly delivery system (LA oral tablets, longer IM injections, SC self‐injections, implants and patches) 6‐ to 12‐monthly clinical visits Alignment with visits for other medical needs
WHERE Service location	ART refills through fast‐track in PHC or decentralized to community settings Clinical visits at primary healthcare	PHC/hospital (with infrastructure for IM injection, cold chain for RPV, management of syringes and needles)	Decentralized—fast track collection at facilities or outside of PHC and hospitals and into communities (e.g. pharmacies, community‐based organizations, mobile vans, home delivery, etc.)	PrEP refills decentralized to community settings Clinical visits at primary healthcare or via telemedicine	PHC/hospital (with infrastructure for IM injection, management of syringes and needles)	Decentralized—fast track delivery at facilities or outside of PHC and into communities (e.g. pharmacies, community‐based organizations, mobile vans, etc.) supported by telemedicine
WHO Service provider (e.g. clinician, nurse, pharmacist, HCW, CHW and peer)	ART refills distributed by lay providers, including peers, pharmacists, CHWs Clinical visits by trained nurses	Trained HCW for IM injections and ART prescribing	Treatment options supporting self‐management: choice of, for example, 6‐ to 12‐month implant; 6 × monthly oral tablets; 6–12 months of a self‐managed SC self‐injection or patch Trained nurse for prescription, implant insertion and monitoring	PrEP refills distributed by lay providers, peers, pharmacists, CHWs and courier Clinical visits by trained HCWs or lay providers supported by clinicians (prescriptions by clinicians)	Trained HCW for IM injections and PrEP prescribing and monitoring	Treatment options supporting self‐management: choice of, for example, 6‐ to 12‐month implant; 6 × monthly oral tablets; 6–12 months of a self‐managed SC or patch Clinical visits by trained HCWs or lay providers supported by clinicians (prescriptions by clinicians)
WHAT Package of services provided	ART refills (& psychosocial support) And at clinical consultations prescriptions, clinical and lab monitoring, OI and other management	Same as current DSD	Same as current DSD + site for implant insertion + product and service integration for other health needs, including STIs, contraception, NCDs and GAHT	PrEP refills, other prevention commodities, HIV self‐test kits, HIV risk and PrEP effective use counselling, PrEP clinical management and monitoring	Same as current DSD	Stipulated minimum package of services (supporting demedicalization) + product and service integration for other health needs, including STIs, contraception, NCDs and GAHT

Abbreviations: ART, antiretroviral therapy; CAB, cabotegravir; CAB/RPV, cabotegravir/rilpivirine; CHW, community‐health worker; DSD, differentiated service delivery; GAHT, gender‐affirming hormonal therapy; HCW, healthcare worker; IM, intramuscular injection; LA, long‐acting; NCDs, non‐communicable diseases; OI, opportunistic infection; PHC, primary health centre; SC, subcutaneous.

^a^ CAB/RPV, lenacapvir.

^b^ CAB‐LA.

^c^ Oral lead in and specifics on loading doses also to be considered.

^d^ Could also consider 6‐monthly SC injection with 6‐ to 12‐monthly clinical review for the treatment of HIV‐1 infection in heavily treatment‐experienced adults with multidrug resistant HIV‐1 infection and not currently available in low‐ and middle‐income countries (initiation oral dosing also to be considered).

### 2.1 Considerations for future positioning and integration into DSD of LAED products for HIV treatment

Globally, an estimated 28.7 million people were accessing HIV treatment by the end of 2021 [18]. In low‐ and middle‐income countries, upwards of 90% of these people are on a daily fixed‐dose combination oral pill that is largely available, affordable and well‐tolerated [19], and are virally suppressed [18].

Accelerated by the availability of TLD, HIV treatment programmes have adopted and implemented DSD [20, 21, 22]. Rather than a “one‐size‐fits‐all” approach, those who are clinically suppressed, or “established on ART,” are eligible for DSD models where the building blocks of their ART refills are separated from their clinical consultations [7]. ART refills have been extended to 3–6 monthly and are available through the four DSD models described in the WHO 2021 consolidated guidelines [7]. Particularly in eastern and southern Africa, a DSD approach to HIV treatment has been widely adopted within national guidance following recommendations from WHO and support from global funders [7, 23, 24]. Implementation data highlight that more than 4.5 million people supported by PEPFAR in 2021 received 6‐month ART refills with an additional 5.7 million on 3‐ to 5‐month ART refills [25]. In South Africa, a cohort of approximately 1.45 million people living with HIV receive their ART refills outside of health facilities and closer to home [26].

Given the high levels of uptake of TLD delivered through DSD models, the target audience and formulation of a future LAED treatment product will require careful consideration. LAED products may be targeted as possible treatment options to specific individuals or populations for whom adherence on daily oral ART is a challenge. Intra‐ and inter‐personal challenges affecting adherence and viral suppression include struggles with daily pill taking, fear of disclosure (driven by both external and internalized stigma) and different stages of the life course (e.g. adolescents, during pregnancy and breastfeeding, elderly) [27, 28]. In addition, lower rates of viral suppression are consistently observed in specific populations, such as children and adolescents [18], despite advances in regimens and innovations in service delivery. LAED products could directly address some of these challenges and support improved outcomes in these specific populations, including decreased rates of perinatal transmission.

There is also increasing evidence of the challenges faced among those living with HIV who are highly mobile and may not be able to carry large supplies of ART or reliably adhere to therapy, collect refills or attend appointments even if they intend to do so, all of which is associated with risk of poor outcomes and development of drug resistance whether using daily or long‐acting therapies [29, 30, 31]. LAED products that support increased self‐management—longer oral tablet regimens, patches or self‐injections—and have some forgiveness in their dosing schedules could offer a better alternative to daily oral tablets for this vulnerable population.

For people living with HIV who are established on treatment, DSD using daily oral pills has enabled less frequent clinic visits with some settings providing an annual clinical visit and 6‐monthly refills. Currently available LAED treatment products are delivered by intra‐muscular injections. These require private and confidential space, the ability to safely discard needles and syringes, and additionally must be staffed by trained and qualified administrators of the injection. In addition, the cabotegravir/rilpivirine long‐acting formulation has a cold‐chain requirement further complicating its administration. It is also a non‐nucleoside reverse transcriptase inhibitor associated with increased risk of both pre‐existing drug resistance among likely clients, including adolescents, and of acquired drug resistance if clients are not adherent to the repeat injection schedule that will necessitate a frequent interaction with the health system. Until such LAED products are available that can match the current frequency of clinical visits (long‐acting (LA) oral pills, implants or patches), DSD programmes must innovate to maximize the benefits of available injectable LAED options for treatment. This may include adapted policies supporting task sharing and decentralization of intramuscular injections, including training of community and potentially lay cadres, and delivery via fixed outreach or mobile clinics. For countries already leveraging private pharmacy networks for the distribution of oral ART tablets [32, 33], expansion of the partnerships to include the distribution of other formulations and injection administration of injectables could be considered.

Costs, acceptability and feasibility—for the health system, healthcare providers and clients—need to be considered along with the challenges and cost implications of the supply chain (including cold chain if required). The benefits of co‐formulations, especially with contraception, are increasingly critical. Reducing the frequency of health facility visits has increased the risk of reducing contraceptive coverage and increased unintended pregnancies among those on short‐acting intramuscular injectable contraception, requiring focused co‐formulation product development to ensure both ART and contraceptive coverage.

### 2.2 Considerations for future positioning and integration into DSD of LAED products for HIV prevention

PrEP for people at substantial risk of acquiring HIV has been recommended by WHO since 2015 [6]. Compared to the successful scale‐up of HIV treatment, access to PrEP has fallen far below global targets [34, 35]. The majority of PrEP scale‐up has been in high‐income countries, predominately among gay men and other men who have sex with men [18]. Until recently, the only PrEP option was oral PrEP, which is known to be associated with mild and self‐limiting side effects that can result in early discontinuation [36]. In low‐ and middle‐income countries, progress towards improving PrEP uptake has been challenged by poor access, the limited choice of PrEP method and considerable costs, including to the client. Further, effective use of PrEP has been limited with high rates of discontinuation [37, 38, 39]. Stigma and challenges with disclosure of PrEP use to partners and family has been cited as an underlying reason for the poor uptake and continuation of PrEP services [40, 41].

In response to COVID‐19, adaptations to PrEP service delivery resulted in increased flexibility with trends towards demedicalization, simplification and integration within other health services [42], provision of virtual support and DSD for PrEP aligning with client preferences [35, 43, 44, 45]. As a result, the number of people on oral PrEP doubled between 2020 and 2021 from 800,000 to 1.6 million [18]. This suggests that DSD models have the potential to increase PrEP access. New technical considerations released by WHO in 2022 provide guidance on differentiated PrEP service delivery, including community‐based and virtual support, longer durations of PrEP refills, reduced clinical visits for PrEP users with support provided by peers and increased use of HIV self‐testing to enable PrEP continuation [46].

To support the scale‐up of PrEP, LAED products could be offered to all PrEP clients as part of a PrEP menu aimed at overcoming specific individual and health system barriers by optimizing DSD features, such as access to services outside of the facility and minimal engagement with health professionals [47]. This differs from the approach discussed for treatment, where LAED products may be more appropriate for specific populations. Offering an informed choice of PrEP products, including LAED, must be presented to reflect relative efficacy but also take into consideration user preferences that may impact on stigma, changing risk exposures, challenges to travel to a facility and longer‐term adherence.

Key considerations for the choice of PrEP agent could include the discreteness of LAED products that may overcome the stigma faced by people taking daily pills [48, 49] as well as adherence challenges. Results from the HIV Prevention Trials Network studies 083 and 084 of comparing injectable cabotegravir and daily oral tablets illustrate that adherence to LAED dosing may be easier than daily pill taking in populations at substantial risk of HIV acquisition [50, 51]. LAED products that enable increased self‐management, such as self‐injectables, long‐acting oral tablets or patches or 6‐ to 12‐monthly intramuscular injectables, will also address challenges of frequent clinical visits and daily adherence.

The duration of HIV risk or vulnerability will also impact the choice of PrEP product and DSD model to deliver it. HIV risk is not a constant and this variability is an important factor in determining PrEP method choice and the ideal duration of PrEP needed and wanted. For some who have longer‐term more continual HIV acquisition risk, a longer‐acting (6–12 monthly) injection may be preferable. For others, who experience periods of vulnerability, a self‐managed long‐acting oral pill or patch that provides prophylaxis for a month may be sufficient to cover the period of vulnerability and, therefore, be preferable to a longer‐acting injectable that requires administration by a healthcare provider. Counselling needs to be framed around reasons for stopping PrEP relative to ongoing risk, empowering self‐managed PrEP use and ensuring that users understand the risks associated with missed doses with respect to emergent infection and theoretical risk for resistance during this period. Capacity building for healthcare workers to adequately assess this risk and offer choice based not only on efficacy but also on user preferences in terms of formulation and how that impacts on the where and when of PrEP delivery will be essential.

Considering integration into DSD for PrEP services of currently available long‐acting products, the DPV‐VR could already be provided as a multi‐month refill as it does not require a cold chain for storage and is self‐administered. However, as for treatment, currently available long‐acting injectable PrEP requires a 2‐monthly intra‐muscular injection. Therefore, the same innovations will be needed, including adapted policies supporting task sharing and decentralization of intramuscular injections.

In addition to considering the changes in risk, other essential components of the service package—such as HIV self‐testing, contraception commodities, gender‐affirming hormonal treatment and possibly other self‐test products such as those for sexually transmitted infections (STIs) and viral hepatitis—should be optimized to enable increased and virtual delivery and re‐initiation of PrEP [52]. There is an opportunity to consider how best to integrate other sexual and reproductive health services in this self‐care model, and it is likely PrEP users will place a premium on comprehensive sexual health services, not just a reduced risk of HIV acquisition. There is also a research need to better understand self‐testing in the context of LAED given the challenges in diagnosis.

As LAED PrEP products are introduced, the choice remains a priority. The recent increase in the rate of PrEP users that has coincided with DSD approaches to PrEP implementation suggests that there is still considerable potential growth with daily oral PrEP if the DSD approach is more broadly supported in policy and implementation scaled. Introduction of the combined PrEP and contraceptive products is under investigation, including a dual prevention pill [53], with a 2024 anticipated approval [54, 55], and co‐formulated vaginal ring products [56]. It may be that developing a user base for any HIV prevention product will smooth the path for future LAED products that may have more optimal characteristics [57]. As LAED products are introduced, it will be important that they are offered as part of a menu of PrEP options and not deemed preferential just because they are long‐acting. Shared decision‐making with the client reflecting the relative efficacy of the product alongside issues of stigma and confidentiality will continue to be critical to scaling up of PrEP services.

Finally, and critically, prevention programming requires finances and resources—funding PrEP is challenging within the HIV and global health space [18]. New LAED products have the potential to expand the PrEP market for both daily oral PrEP and future LAED products with longer durations that are more easily administered and difficult trade‐offs may have to be made.

## 3 CONCLUSIONS

There are exciting new options for LAED products for HIV treatment and prevention, both in the pipeline and receiving regulatory approval. The success of future LAED products will be determined by “how” their delivery improves on what is currently offered by daily oral therapies recognizing the balance between some clients preferring reduced facility visits versus those who will accept additional clinic visits for the benefits of reduced pill burden and possible reduction of stigma. For HIV treatment, daily oral therapies already offer a high standard of care on metrics, such as acceptability, effectiveness, feasibility and cost, all of which have been further optimized through the use of DSD. However, there are specific populations for whom the current regimen presents challenges that may be overcome with future LAED products. For HIV prevention, however, where method choice is a priority and delivery of oral PrEP to date has been overmedicalized, LAED products that better align with the concepts of DSD may prove to be very acceptable to all stakeholders. Funding for the future integration of LAEDs and ongoing advocacy for a sustainable and affordable supply compared to existing treatment and prevention options will be essential. For any LAED product, it is critical to emphasize the use of implementation science metrics to define and prioritize the challenges that a new product seeks to address from the perspective of the client, the provider and the health system.

## COMPETING INTERESTS

The authors declare no competing interests.

## AUTHORS’ CONTRIBUTIONS

AG, LW and HB conceived of the manuscript. SD‐M, PE, KG, MM, KN, NJO, MP, WV and MV provided inputs on the initial outline. MV drafted the initial table and AG drafted the initial manuscript. All authors provided inputs on the draft manuscript and approved the final submission.

## FUNDING

AG is supported by the Bill & Melinda Gates Foundation (INV002610).

## Data Availability

There are no primary data in this submission and, therefore, this is not applicable.
